# Distal Spike Initiation Zone Location Estimation by Morphological Simulation of Ionic Current Filtering Demonstrated in a Novel Model of an Identified *Drosophila* Motoneuron

**DOI:** 10.1371/journal.pcbi.1004189

**Published:** 2015-05-15

**Authors:** Cengiz Günay, Fred H. Sieling, Logesh Dharmar, Wei-Hsiang Lin, Verena Wolfram, Richard Marley, Richard A. Baines, Astrid A. Prinz

**Affiliations:** 1 Department of Biology, Emory University, Atlanta, Georgia, United States of America; 2 Department of Biomedical Engineering, Georgia Institute of Technology and Emory University, Atlanta, Georgia, United States; 3 Faculty of Life Sciences, University of Manchester, Manchester, United Kingdom; The Krasnow Institute for Advanced Studies, UNITED STATES

## Abstract

Studying ion channel currents generated distally from the recording site is difficult because of artifacts caused by poor space clamp and membrane filtering. A computational model can quantify artifact parameters for correction by simulating the currents only if their exact anatomical location is known. We propose that the same artifacts that confound current recordings can help pinpoint the source of those currents by providing a signature of the neuron’s morphology. This method can improve the recording quality of currents initiated at the spike initiation zone (SIZ) that are often distal to the soma in invertebrate neurons. *Drosophila* being a valuable tool for characterizing ion currents, we estimated the SIZ location and quantified artifacts in an identified motoneuron, aCC/MN1-Ib, by constructing a novel multicompartmental model. Initial simulation of the measured biophysical channel properties in an isopotential Hodgkin-Huxley type neuron model partially replicated firing characteristics. Adding a second distal compartment, which contained spike-generating Na^+^ and K^+^ currents, was sufficient to simulate aCC’s *in vivo* activity signature. Matching this signature using a reconstructed morphology predicted that the SIZ is on aCC’s primary axon, 70 μm after the most distal dendritic branching point. From SIZ to soma, we observed and quantified selective morphological filtering of fast activating currents. Non-inactivating K^+^ currents are filtered ∼3 times less and despite their large magnitude at the soma they could be as distal as Na^+^ currents. The peak of transient component (NaT) of the voltage-activated Na^+^ current is also filtered more than the magnitude of slower persistent component (NaP), which can contribute to seizures. The corrected NaP/NaT ratio explains the previously observed discrepancy when the same channel is expressed in different cells. In summary, we used an *in vivo* signature to estimate ion channel location and recording artifacts, which can be applied to other neurons.

## Author Summary

The study of ion channels is essential both for understanding normal brain function and for finding drug targets to treat neurological disease. Traditional experimental techniques remain challenging for recording ion channel currents accurately because of their locations in the neuron. Computer modeling of the three dimensional structure of neurons can provide a correction estimate for the measurement error introduced by neuronal membranes. To achieve this, we developed a modeling approach to localize, and correct for, distant ion channels. We demonstrated this approach by constructing novel computer models of an identified insect motor neuron, which provides a powerful model for studying the central nervous system. Through the study of electrical activity and genetic manipulations, it has been found that the persistent sodium current contributes to seizure. By modeling three dimensional structure, we were able to predict the location of these currents in the neuron, which were more distal than expected. Localizing sodium channels allowed us to predict their properties at origin, which favorably matched isolated recordings of these channels in more compact cells. This result is important in validating the use of heterologous compact cells to study insect sodium channels, and also demonstrates the usefulness of the presented modeling approach for studying channel physiology more generally.

## Introduction

Space clamp errors, a major problem in neuronal recordings [1], can be estimated using model simulations [2]. Neuron models with morphological detail have also been useful for estimating other properties that are difficult to measure experimentally [3]. Along with space clamp artifacts, membrane filtering properties contribute to a neuron’s characteristic *in vivo* firing properties, which can also be simulated with a morphologically reconstructed model. If ion channel biophysics can be estimated *a priori*, then the success of simulations requires the correct placement of ion channels in the morphology. Here, we propose a simulation method for identifying ion channel distributions that match *in vivo* output which, in turn, predicts *in vivo* channel localization. Furthermore, the ion currents in the resulting model can serve to simulate filtering and space clamp artifacts. This is important for quantifying measurement errors when characterizing ion currents for the study of normal and pathological neuronal function that is correlated with neuronal morphology. We applied this method to study distally generated currents in a motoneuron of the fruitfly *Drosophila* *melanogaster*.

*Drosophila* has recently emerged as a powerful model for studying neuronal physiology through genetic screening [4] and electrophysiology of its identified neurons. *Drosophila* was pivotal for characterizing ion channel biophysics and their role in neuronal function. Voltage-gated potassium ion channels, originally discovered in *Drosophila*, were found to be structurally and functionally homologous to those in mammals [5]. These channels were characterized from diverse sources including generic neuron types and muscle cells maintained in culture [6–8], heterologous expression [9], and in growth-arrested embryonic neuroblasts [10]. Perhaps not surprisingly, these varied approaches have resulted in inconsistent parameters for the same channels. Computational models that incorporate average values [11], whilst of value, probably yield unphysiological outcomes [12]. A complementary approach is to model individual identified neurons, which often differ significantly in their electrophysiological properties [13, 14]. The recent development of *in vivo* electrophysiology applied to *Drosophila* identified motoneurons [13, 15, 16] allowed us to characterize channels based primarily on experimentally-derived data from the larval aCC motoneuron (also termed MN1-Ib). However, spatial errors must be elucidated to establish that these characterizations are sufficiently accurate.

Use of the aCC motoneuron for our studies is particularly relevant because this neuron is without doubt one of the best studied in terms of channel electrophysiology [13, 15, 17–20]. It is also relevant for studying channelopathies, such as the role of sodium channels in seizure disorders [21]. *Drosophila* expresses only one variant of the sodium channel (DmNa_v_), which is responsible for both transient (NaT) and persistent (NaP) current components [22]. Recently, the kinetics of this channel, in aCC, was studied in both wildtype and in genetic seizure (bang-sensitive) mutants [23]. As a result, seizure tendency has been linked to increased NaP in seizure mutants through both recordings from heterologous expressions and *in vivo* [20]. However, there is a puzzling discrepancy between the ratio of NaP/NaT in the two conditions: the *in vivo* ratio being much higher brought into question the correctness of heterologous expression experiments. While more accurate recordings from heterologous expression in electrotonically compact *Xenopus* oocytes could not predict firing activity changes, *in vivo* recordings in aCC were also complicated by neuronal homeostatic and other compensatory mechanisms that likely act to regulate firing properties to maintain consistency of spiking activity in a mutant genetic background [17, 20, 24]. To isolate only the contribution of NaP, we have previously constructed a computational neuron model from individual channel biophysics with a Hodgkin-Huxley type formalism [20]. Using this model, we have confirmed that increasing NaP raises the firing rate. However, the model did not include morphological characteristics that undoubtedly contribute to the impact of ionic conductances in this neuron. Without including morphological detail in this neuron model, discrepancies between *in vivo* and heterologous expression could not be explored.

In *Drosophila*, single-compartment models were used to simulate neurons and study firing properties [11, 15]. Models of *Drosophila* neurons were morphologically reconstructed without active ion channels for performing passive analysis [26–28]. Using the well-studied anatomy of aCC [29–31], we predicted the spike initiation zone (SIZ) location by computer simulation of its reconstructed morphology. While establishing the membrane filtering properties with the SIZ localization, we studied two aspects: (1) the dendritic distribution and density of K^+^ channels in aCC and (2) the effect of filtering on the NaP/NaT ratio at the soma versus the SIZ.

## Results

Our proposed method for making anatomical predictions requires establishing many parameters of aCC’s physiological properties. Instead of determining all of these parameters in a complex morphological model, we progressively constructed a series of models that each established important properties. We started from a basic model with a single-compartment, then moved to one with two compartments, and then moved to one with a full morphological reconstruction. Our first step in this process was to determine biophysical properties of aCC ion channels that replicate basic firing characteristics.

### Minimal biophysical model neuron replicates firing of larval aCC motoneuron

**Fig 1:**
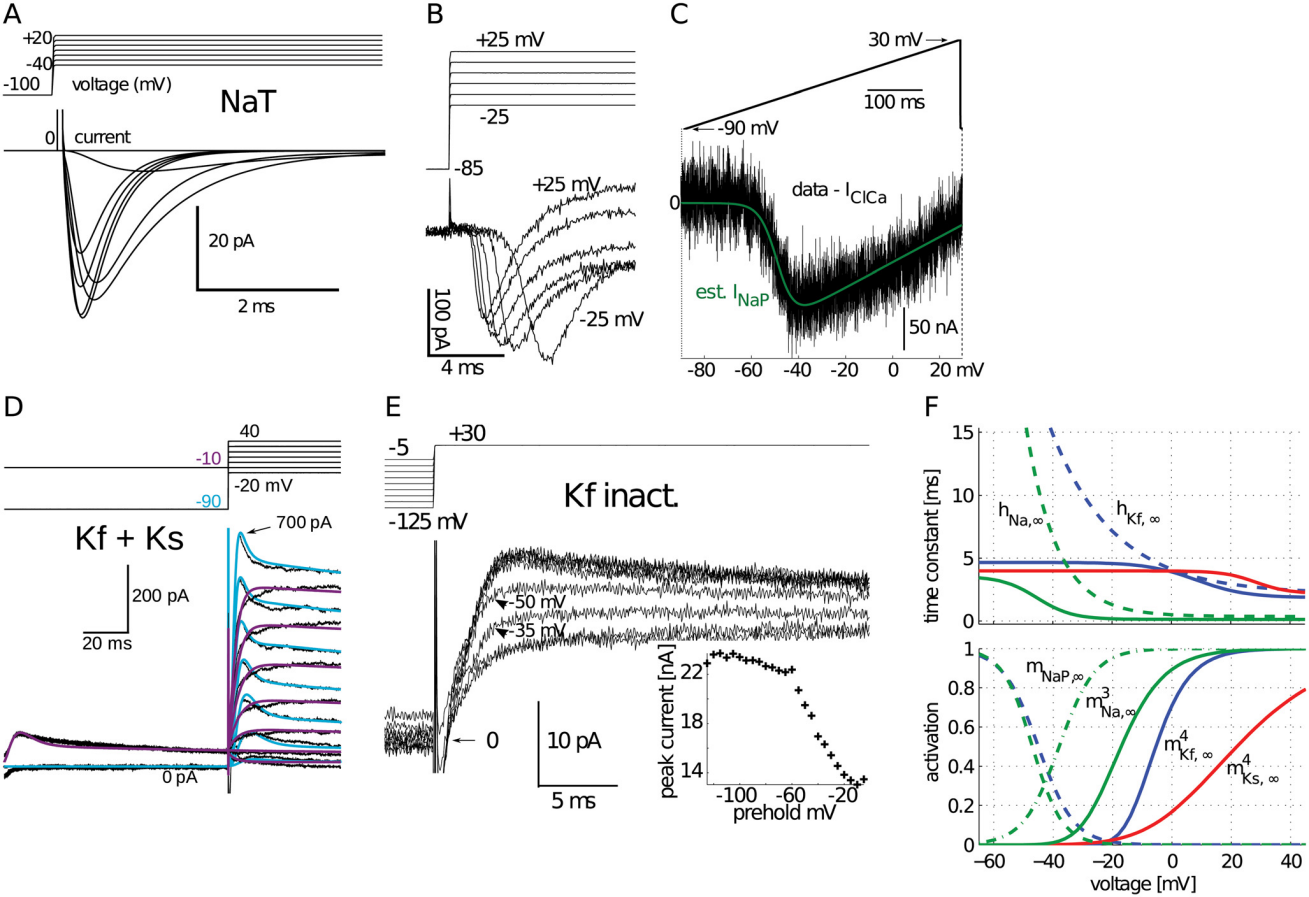
Ionic currents modeled from identified larval aCC motoneurons. (**A**) Simulated voltage clamp Na^+^ current to replicate previously published responses by [66] recorded in cultured *Drosophila* motoneurons. (**B**) Isolated Na^+^ current recorded under voltage clamp from a 3rd instar aCC motoneuron was slower with large onset delays caused by poor space clamp. (**C**) Persistent Na^+^ current (NaP) estimated from response to voltage ramp applied to a *Xenopus* oocyte, after subtracting endogenous current (see Methods). (**D**) Slow (Ks) and fast (Kf) K^+^ currents modeled from voltage clamp recordings, simulated as a single cumulative trace (smooth lines). Together they were simulated with two voltage clamp protocols shown superimposed, one with a preholding voltage at −10 mV (purple) to inactivate the fast component and another one preholding at −90 mV (light blue). (**E**) Voltage clamp recordings in presence of Cd^2+^ for measuring K^+^ channel inactivation by using a 500 ms varying-level preholding voltage. Peak current plotted in the inset shows voltage-dependence of inactivation. (**F**) Summary of modeled currents (also see Table 5.); voltage dependence of time constants and the gates of activation (m) and inactivation (h). The Kf current model also included a second, slow inactivation with a time constant of 135 ms (not shown).

To maintain faithfulness to the identity of aCC, we obtained a complete set of biophysical parameters as directly as possible using different sources: from *in vivo* recordings of aCC for its slow and fast K^+^ currents; from heterologous expression of the DmNa_v_ sodium channel for the persistent Na^+^ current component, and from cultured fly neurons for having a description of the transient Na^+^ current component without being subject to space clamp errors (see Methods and Fig 1). The resulting biophysical parameters of the Hodgkin-Huxley type ion channels were represented with voltage-dependent functions for activation, inactivation, and time constant variables (Fig 1F).

**Fig 2:**
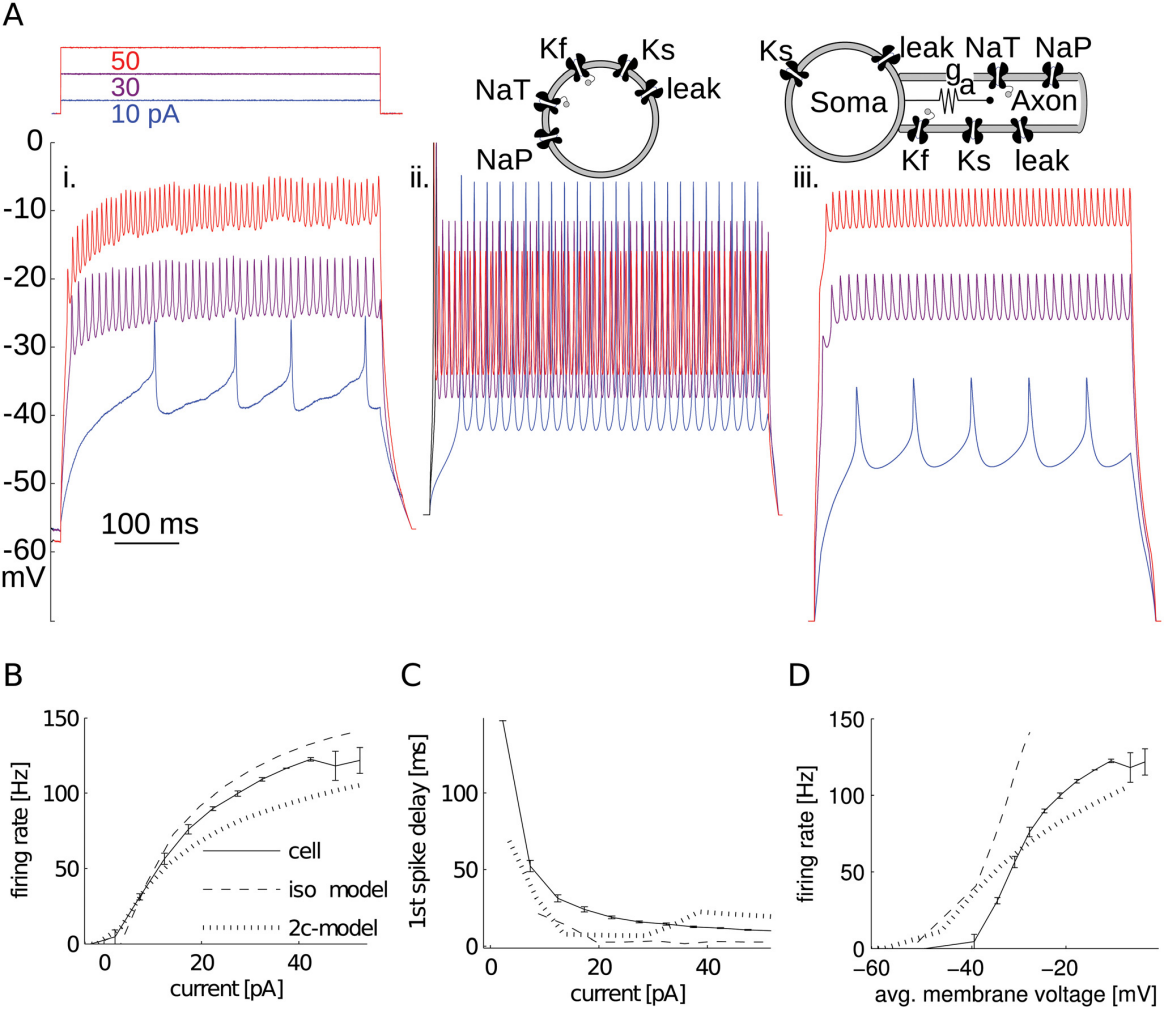
Isopotential and two-compartment models compared to recordings. (**A**) Third instar aCC motoneuron recording (i.) in presence of 0.2 mM Cd^+2^ to block Ca^+2^ channels missing from models. Voltage response to three current injection levels. Isopotential (ii.) and two-compartment (iii.) models’ voltage responses to same current injection. (**B**) Recorded and simulated firing responses to current injection were similar. Firing rate was calculated as the inverse of mean interspike interval (ISI). iso-model: isopotential model; 2c-model: two-compartment model. (**C**) Models also approximated aCC motoneuron delay to first spike in response to current injection. (**D**) The firing rate-membrane voltage (f-V) response of only the two-compartment model qualitatively mimicked the inter-spike voltage depolarizations that appeared for high firing rates.

Our biophysical model of aCC was primarily constructed to study action potential firing. To keep it minimal, we omitted Ca^2+^ and Ca^2+^-dependent channels because aCC continues to generate action potentials even when Ca^2+^ currents are blocked (Fig 2Ai). To test whether aCC’s channels need a spatial component to replicate firing properties, we first simulated a minimal set of biophysical channels together in a single-compartment (isopotential) model neuron (Fig 2Aii). This model successfully mimicked aCC’s characteristic firing rate-current (f-I) relationship (Fig 2B). It was also able to exhibit large delays for small current injections, which is characteristic to these motoneurons (Fig 2C). The firing rate of aCC, during a current stimulus, is relatively stable with a coefficient of variation (CV) of 0.076 (10 pA current stimulus, 16.11 pF cell capacitance, firing rate at ∼ 35 Hz). The model had the same feature with a similar CV = 0.002 (10 pA and 50 Hz). The non-zero value of the CV in the model arises from the spike frequency adaptation characteristic caused by channel dynamics. However, this simple isopotential model neuron failed to match the observed spike amplitude and depolarized inter-spike membrane potential in response to current injection (compare 2Ai and ii).

### Modeling voltage depolarizations and spike attenuation requires a distal compartment

Like other larval *Drosophila* motoneurons, aCC has a characteristic electrical response to current injection characterized by a depolarized inter-spike membrane voltage, small spikes, and variable delay to first spike [13]. The depolarization of the inter-spike membrane voltage creates an offset from resting that increases with level of injected current. This voltage offset and small spikes could be explained by the series resistance between the recording electrode, placed at the soma, and distal region with spike-generating Na^+^ channels commonly found in invertebrate neurons. Lacking this spatial dimension, the isopotential model was unable to replicate the voltage offset and small spikes (Fig 2Ai–2Aii). Changes in none of the isopotential model parameters resulted in creating voltage offsets without pushing its input resistance to unphysiological values or losing the ability to maintain firing. The voltage offsets could be replicated in the model neuron, however, when a second compartment was added (Fig 2Aiii).

In the two-compartment model neuron, fast active conductances were placed in the distal compartment, which is consistent with aCC’s known physiology [15]. This model was able to reach similar membrane voltage offsets in response to injected current (Fig 2D), whilst still maintaining similar spiking rate (Fig 2B) and delay (Fig 2C) characteristics. Two-compartment models have been successfully used for modeling other invertebrate neurons [32, 33], and similarly here, it hinted that an important role in determining response properties is played by channel placement and morphology. However, because of its simplicity, this model only provided a rough estimate of ion channel placement. Although multicompartmental models at intermediate levels of complexity could be employed [34, 35], making an anatomical prediction of the SIZ location required a fully reconstructed morphology.

### Using a reconstructed morphology to study ion channel localization

**Fig 3:**
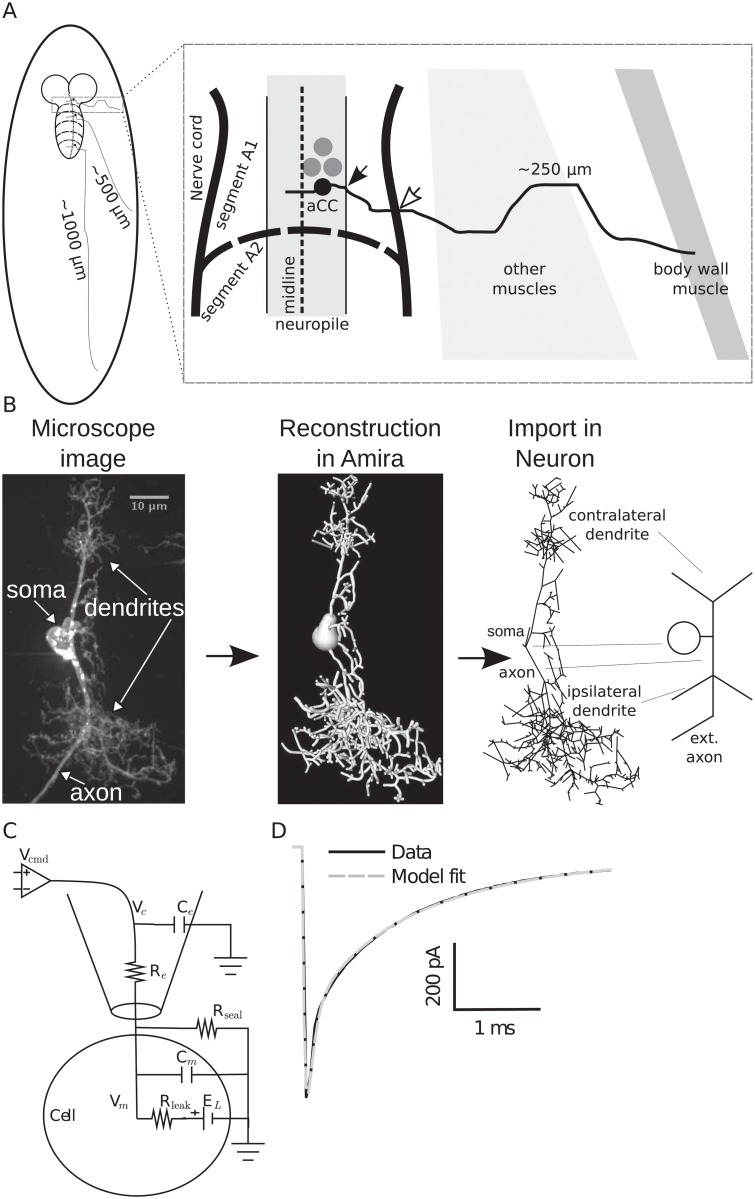
Morphological reconstruction of the 3rd instar aCC motoneuron. (**A**) Schematic muscle projections of aCC motoneurons based on their location in the nerve cord segment. (**B**) Stack of microscope images was reconstructed using Amira software (Visage Imaging GmbH, Berlin, Germany) and then imported into the Neuron simulator [72]. The rightmost schema indicates the major morphological components in an idealized depiction. “ext. axon” indicates the missing extended axon from the reconstruction (not drawn to scale). (**C**) Equivalent circuit of the measured passive properties including an electrode model for voltage clamp. (**D**) Passive response to voltage-clamp step to −90 mV from a holding potential of −60 mV is simulated in Neuron with the fitted parameters (see Table 2).

The exact location of distal active currents predicted by the two-compartment model on aCC’s anatomical morphology and the effect this has on the observed channel currents are unknown. The aCC anatomy consists of two dendritic arbors, the smaller of which crosses the midline [36]. By contrast, the larger primary neurite (axon) exits the nerve cord ipsilaterally to enter the muscle field (marked by empty arrow in Fig 3A) and projects through the intersegmental nerve to dorsal body wall muscle (DA1). It is known that larval aCC contains TTX-sensitive Na^+^ channels and that these channels are essential for action potential generation [15]. The distribution of Na^+^ channels and location of the SIZ in aCC are unknown. To simulate possible localizations, we used a reconstructed morphology of a 3rd instar aCC motoneuron derived from confocal microscope image stacks [31] and developed an electrical model (Fig 3B). The morphological reconstruction was limited to the two dendritic arbors, and did not contain the complete extent of the axon (which can be relatively long, see Fig 3A). Properties of different anatomical sections in this morphology were calculated (Table 1).

**Table 1:** Morphological statistics of example aCC motoneuron, divided into sections.

Section	L [*μ*m]	Diam. [*μ*m]	Area [*μ*m^2^]	*R*_*i*_ [MΩ]	*c*_*m*_ [pF]
Soma	6.98	5.50	120.56	0.06	0.60
Axon	37.61	1.14	134.11	7.46	0.67
Ipsilateral dendrite (w/o axon)	959.10	0.76	2292.49	423.27	11.46
Contralateral dendrite	365.99	0.72	837.85	176.09	4.18
Extended axon	570.00	0.71	1275.49	1556.01	12.75
*Total*	*1939.68*	*N/A*	*4660.50*	*N/A*	*29.66*

*L*, total length of section branches; Diam., length-weighted equivalent diameter of section; Area, surface area, *R*_*i*_, resistance of from beginning to middle of section; *c*_*m*_, total maximal capacitance of section (does not consider decay of voltage).

Passive parameters of the model were obtained by fitting responses of the morphological model to electrophysiological recordings of hyperpolarizing current steps (see Methods). We developed a method to compensate for measurement artifacts that occur in recordings of these motoneurons because of their high input resistance, which cannot be automatically compensated [7, 13, 18, 19, 37]. To include the dynamical artifacts resulting from these errors, we modeled the passive electrode properties as an additional compartment in our morphological model (Fig 3C). All passive parameters of this model, including the electrode parameters, were fitted to recordings (Fig 3D and Table 2). The passive morphology cable parameters we derived were similar to those reported in *Drosophila* central neurons (see Table 1 in [26]). Establishing the passive electrical properties allowed study of the signal propagation in the morphology with particular reference to distally-generated currents.

**Table 2:** Morphology passive parameters fitted to 3rd instar aCC responses to passive voltage clamp protocol recordings (*n* = 5).

Parameter	Mean±SE
*R*_*a*_ [Ω ⋅ cm]	212.47±3.69
*C*_*m*_ [*μ*F/cm^2^]	0.77±0.02
*g*_leak_ [*μ*S/cm^2^]	37.96±4.38
*g*_seal_ [nS]	0.20±0.03
*C*_e_ [pF]	1.28±0.24
*R*_e_ [MΩ]	41.47±3.88

### Passive morphology is electrotonically compact, but also a strong low-pass filter

**Fig 4:**
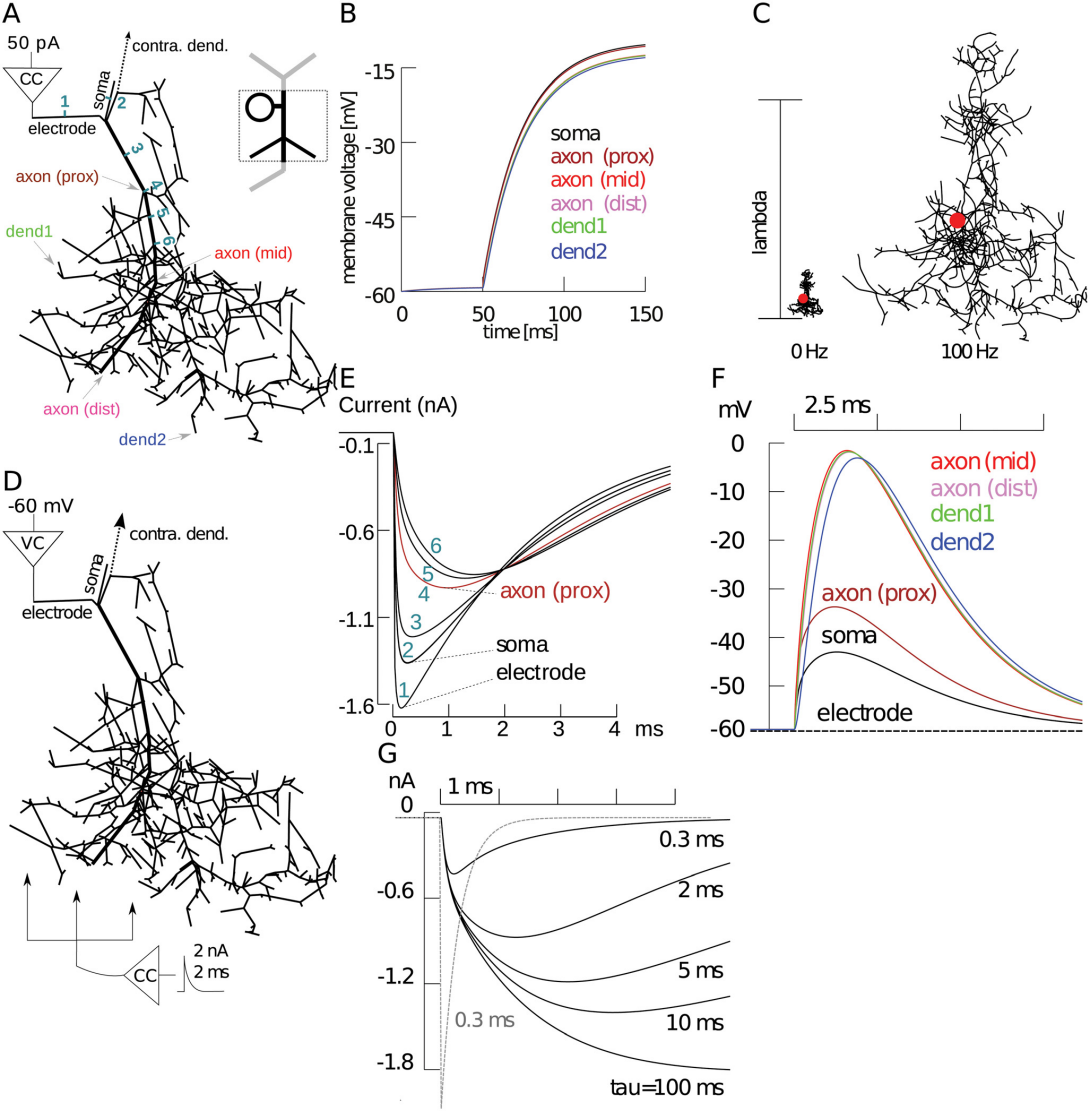
Simulations of the passive morphology showed its compactness and filtering properties. (**A**) Simulation setup for the ipsilateral dendritic field of the reconstructed morphology (thick line is the primary neurite), where a 50 pA current clamp (CC) stimulus was applied at the model electrode compartment. (**B**) Voltage across model morphology locations marked in panel **A** in response to the current stimulus. (**C**) Electrotonic structure of the morphology compared at different stimulus frequencies. Lambda scale bar shows one length constant, at which voltage changes to about 37% of its initial value. For a step input (0 Hz), the length constant was several times larger than the arbor size, meaning voltages will travel across the arbor unimpeded. At a higher input frequency of 100 Hz, the span across the morphology was larger than one electrotonic length. (**D**) Ionic-like currents were simulated on the passive morphology by varying current injection location and responses were recorded holding the soma in voltage clamp at -60 mV. (**E**) Voltage clamp current evoked in response to exponentially decaying stimulus of 2 nA magnitude and 2 ms time constant injected at six locations in panel **A** starting at the electrode, and numbered in aquamarine as they went distally. (**F**) When same stimulus is injected at middle of compartment axon (prox), compartments far from the electrode escaped from the voltage clamp to various degrees. (**G**) By varying the time-constant of the same exponentially decaying stimulus applied to a fixed location axon (prox) in simulated morphology, we showed that the membrane response is filtered dependent on frequency. Dashed gray line shows the fastest (*τ* = 0.3 ms) current stimulus applied distally.

The theory of passive membranes is well understood, especially for regular morphological structures (for a summary, see [38]). For a more complex morphology such as ours, quantifying passive properties requires simulation. A current of 50 pA injected under current-clamp *in vivo* (into 3rd instar aCC with 16 pF) results in fast spiking (see Fig 2Ai). However, we cannot reliably measure the voltage distribution it causes across the entire dendritic morphology, which would affect activation of ionic currents. Morphological properties would dictate the extent to which dendritic inputs (i.e., synaptic inputs), originating from those locations, would influence the soma [39]. Therefore, we simulated the same somatic current injection and studied voltage distribution in the largest part of the reconstructed morphology, the ipsilateral dendritic arbor (Fig 4A). As a result, most of the dendritic arbor reached the same voltage: there was less than 5 mV of difference across the whole arbor (Fig 4B). It is known that attenuation in the reverse direction may not be the same and it was recently found that asymmetric signal attenuation in morphologies is important for excitability [18]. However, in our model, stimulus injected into dendrites caused a depolarization that reached the soma without significant attenuation (Table 3). Neuronal membranes act as low-pass filters [38], and aCC has a distinctively slow time course, which is the result of a combination of its membrane area, branching, and internal resistivity parameters. When injecting at the soma (also see recorded responses in Fig 2Ai), distal voltages peaked after ∼ 50 ms. Therefore, somatic responses were strongly attenuated by the low-pass filter (Fig 4C), whereas dendritic inputs were slightly less attenuated at high input frequencies (Table 3). This asymmetry was expected from previous work [38].

**Table 3:** Symmetry of voltage attenuation in the morphology.

	0 Hz		100 Hz	
Measured at:	elec	distal	elec	distal
dend1	0.95	0.93	0.70	0.65
dend2	0.90	0.92	0.51	0.64
contra tip	0.85	0.95	0.37	0.78
SIZ	0.83	0.65	0.10	0.33

Each row indicates a morphological location for which we simulated the ratio of voltage amplitude at measurement site over voltage at current injection site for two conditions: injecting distally and measuring at somatic electrode (elec), and injecting at the electrode and measuring distally (distal). Experiments were repeated for direct current (DC, 0 Hz) and high frequency (sinusoidal, 100 Hz) stimuli. In addition to the two dendritic locations (dend1 & 2) in the ipsilateral arbor shown in Fig 4A, we also tested the distal-most tip of the contralateral dendritic arbor (contra tip) and the spike initiation zone (SIZ—see Fig 6D).

Somatic recordings of dendritic inputs, and likewise ionic currents, would be subjected to filtering based on the current’s origin. A larger distance on a low-pass membrane would increase the strength of filtering [38]. To quantify the amount of filtering, we simulated ion channel-like current sources at different spatial locations. The soma was voltage clamped at −60 mV (Fig 4D) to observe the clamp current (Fig 4E) and decay of distal voltage changes (Fig 4F). As a first approximation, ion current-like sources were modeled with an exponentially decaying stimulus of 2 nA magnitude and 2 ms time constant, which were injected at locations on the primary neurite (marked with thick lines in Fig 4D). The current was most strongly attenuated when its injection point was moved from the soma to the compartment after the first dendritic branching point (Fig 4E), consistent with effects of branching in cylindrical compartments [38]. When current injection was fixed at this distance, it was sufficient for several distal locations of the neuron to escape from voltage clamp (Fig 4F). As the current source was moved further distally, the rate of attenuation diminished (Fig 4E). This suggests that branching of the ipsilateral dendritic arbor creates strong filtering points at very short distances from the soma of aCC. The low-pass filtering properties of the morphology would also selectively attenuate high frequency activity, such as currents from faster channels, which would also affect our estimates of their locations.

### Fast, Na^+^-like, currents are attenuated more than slow, K^+^-like, currents

In larval *Drosophila* motoneurons, the magnitude of K^+^ currents can be up to ten-fold larger than Na^+^ currents when recorded at the soma [15], and in third instar aCC motoneurons, we show that this represents a three-fold difference (compare Fig 1B to Fig 1D). Partly because of this difference, Na^+^ currents are presumed to originate more distally than K^+^ currents. However, the magnitudes of these currents at their origin are unknown. Using the present model, we asked whether the observed magnitude difference can be explained by aCC’s morphological filtering. With any low-pass filter, a larger attenuation would be observed for high frequency signals (e.g., fast Na^+^ currents) than for low frequency signals (e.g., slow K^+^ currents) [38]. To quantify how filtering might affect current magnitudes, we simulated the same model setup where the soma is voltage clamped and an artificial current source is injected to the primary neurite (Fig 4D). However, this time we tested currents of different activation speeds by changing the time constant of the decaying exponential signal. As a result, we found that the peak of fast currents, like the transient Na^+^ (NaT) current, with a time constant of *τ* = 0.3 ms was attenuated about 3–4 times more than a slow Ks-like current signal with *τ* = 10 ms (Fig 4G). This suggests that the three-fold difference observed in aCC between these currents can be completely accounted for by filtering. This result has a direct consequence for the presumed location of the currents. If some, or all, of the measured differences in current magnitudes in the soma are because of filtering, Ks currents may be originating further than previously assumed, but filtered less. To confirm these results, we simulated possible Ks current localization using a more realistic current source by simulating the Hodgkin-Huxley type Ks current we modeled earlier from biophysical parameters.

### Delayed rectifier Ks channels cannot be highly expressed near the soma

**Fig 5:**
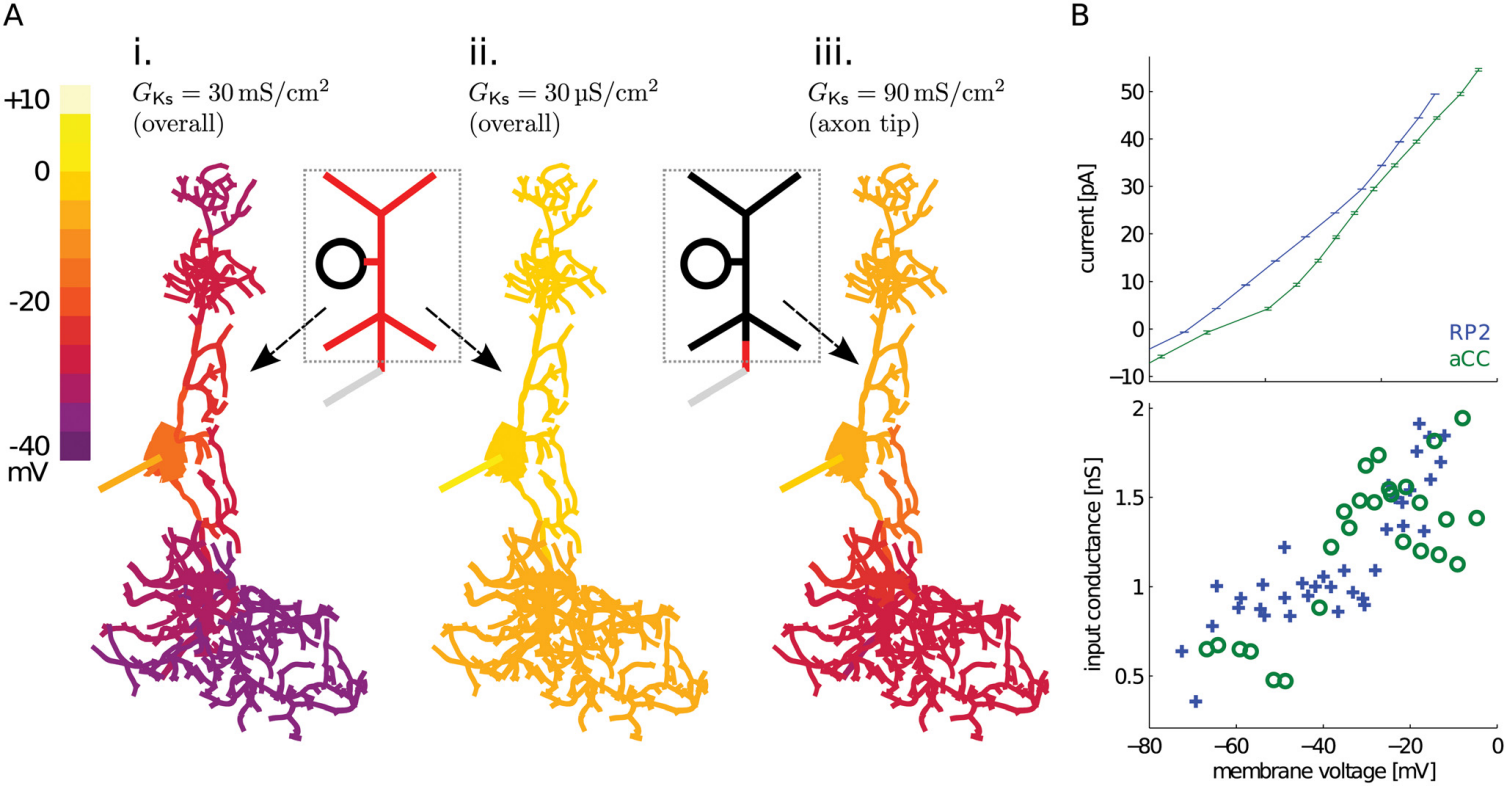
Simulated and observed evidence for delayed rectifier Ks channel activity and distribution. (**A**) Simulating voltage clamp (VC) to 0 mV at the soma and observing voltage distribution over the morphology reflected effects of different Ks channel distributions, whose locations were indicated with red on the schematic in the insets. **i**. When a large Ks channel density of *G*_Ks_ = 30 mS/cm^2^ was applied uniformly to the cell (total *g*_Ks_ ≃ 1*μ*S, slightly larger than the two-compartment model total *g*_Ks_ = 700 *nS*), VC affected only the soma. **ii**. The voltage could spread through the rest of the cell only when Ks density was reduced by three orders of magnitude, to *G*_Ks_ = 30 μS/cm^2^ (total *g*_Ks_ ≃ 1 nS). **iii**. Expression of an even higher density of *G*_Ks_ = 90 mS/cm^2^ in only the most distal 8 main neurite compartments (over an area of 43.37 μm^2^, giving a total *g*_Ks_ = 390.33 nS), while keeping the Ks level in the rest of the cell same as in Panel **ii**, confined voltage clamp to the soma and contralateral neurites. (**B**) Current clamp recordings, in the presence of Cd^2+^ as before, hinted at low somatic Ks density in example larval motoneurons. Mean and standard error of voltage change with input current (I-V curve) between aCC and RP2 motoneurons were similar (top). Slopes of the individual data points in the I-V curves (showing input conductance) were also similar (bottom; using markers instead of lines). The input conductance at the soma only increased by about 1 nS in the presumed Ks activation voltage range.

Our two-compartment model suggests that electrophysiological features of aCC can be mimicked when Ks currents are localized together with NaT currents at a distal SIZ. But Ks current was also observed close to the soma in invertebrate systems [41] and in the dissociated somata of larval motoneurons (R. Baines, unpublished data). The presence of Ks currents can be detected because they reduce the observed cell impedance that affects voltage clamp quality [2, 42]. Therefore, we simulated somatic voltage-clamp to investigate the localization of biophysically-accurate Ks channels (see Fig 1D and Methods), in three different distributions over the morphology (Fig 5).

We tested the effect of Ks current on space clamp by voltage clamping the soma to 0 mV, much above the resting membrane potential. Based on our two-compartment model (see Fig 2 and Methods), a Ks conductance (*g*_Ks_) of 700 nS or more is necessary to generate realistic spiking. If a total of *g*_Ks_ = 1μS was uniformly distributed over the morphology, we found that space clamp was limited to near the soma (Fig 5Ai). This outcome is unrealistic because it would prevent voltage clamping any distal SIZ (e.g., experiments shown in Fig 1) and also abolished the characteristic voltage offset seen in current injection experiments. Therefore *g*_Ks_ near the soma must be lower, but how low must it be?

Ks currents near the recording site at the soma can also be detected in current clamp [42]: observed input conductance would increase at holding potentials above −30 mV where Ks conductances activate (see Fig 1F). In this model configuration (Fig 5Ai), the input conductance increased from 1.42 nS at −60 mV to 12.50 nS at −30 mV. Contrast this ∼ 10 nS increase with example current injection experiments from aCC and another similar motoneuron, RP2, which showed much smaller (∼ 1 nS) increases in input conductance (Fig 5B). When a uniform distribution of this reduced observed Ks density was simulated, space clamp was able to reach more distal locations of the morphology (Fig 5Aii), and the model input conductance measured at −30 mV was also reduced to 1.25 nS. But such low Ks density alone cannot support the observed firing activity of aCC. The SIZ must have a high Ks density co-localized with NaT currents, which can be simulated by placing Ks channels at the distal tip of the primary neurite in high density, and low-density in the rest of the model. This rescued the voltage clamp at the soma and in the contralateral dendritic tree, but not in the ipsilateral dendritic tree and distal neurite (Fig 5Aiii). The model had an input conductance of 3.33 nS at −30 mV, which is close to the observed peak input conductance of ∼ 2 nS (Fig 5B). This scenario is also consistent with our prediction that observed high levels of Ks currents can be misleading because of filtering, and that this current probably originates from distal locations similar to NaT currents. Next, we investigated the exact localization of the SIZ by simulating spiking activity.

### Model predicted Na^+^-K^+^ channels to be surprisingly far from the soma

**Fig 6:**
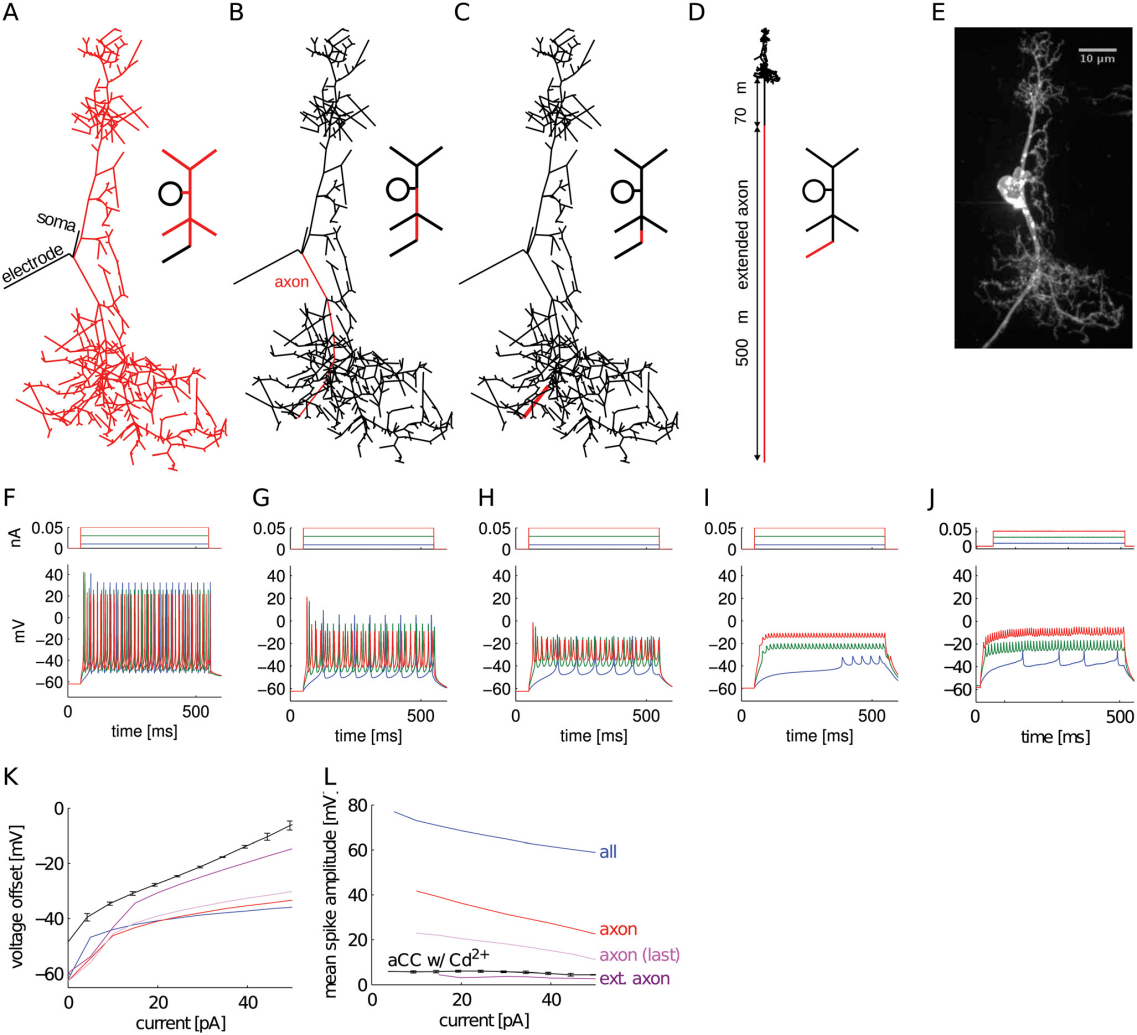
Different distributions of NaT/Ks channels on the constructed morphology predicted SIZ location that can reproduce firing activity characteristics. (**A**-**C**) Skeletal model reconstructions showing high density of NaT/Ks channels in red. (**D**) Axon extension with NaT/Ks channels placed on the distal segment. (**F**-**I**) Simulated responses to the three-step current clamp of each model corresponding to channel distributions in panels A-D. (**E**, **J**) Morphology and recorded responses of aCC to same input current levels, repeated from Fig 2Ai for comparison. Change of voltage offset (**K**) and spike amplitude (**L**) with current injection compared between the NaT/Ks placements in (**A**-**D**) and the biological recording (aCC w/ Cd^2+^).

To determine the SIZ location, we determined which placements of high-density, spike-generating, Na^+^/K^+^ channels would yield the current clamp response observed at the soma. To this end, we tested the effect of placing NaT/Ks channels (see Table 4 for conductance densities) in different regions of the reconstructed morphology (Fig 6A–6C) and simulated neuron response to current clamp stimulation (Fig 6F–6H).

**Table 4:** Other parameters varied to obtain realistic firing activity from morphological models with different NaT-Ks channel distributions.

Na-K channels	*g*_Ks_ [mS/cm^2^]	*g*_NaT_ [mS/cm^2^]	*g*_NaP_ [*μ*S/cm^2^]	*g*_leak_ [*μ*S/cm^2^]	*g*_leak, elec_ [*μ*S/cm^2^]
All	55	30	0.1	3.28 × 10^−2^	627
Ipsilateral dendrite	55	30	0.1	3.28 × 10^−2^	627
Recon. axon only	400	180	0.1	3.28 × 10^−2^	627
Recon. axon last seg.	3,000	1,000	0.5	3.28 × 10^−2^	627
70 *μ*m into ext. axon	850	240	110	1	777

Recon: reconstructed; elec: electrode.

Similar to the isopotential model (Fig 2), placing the NaT/Ks channels throughout the morphology (Fig 6A) resulted in spikes that are taller than the observed 10 mV magnitude under *in vivo* conditions (Fig 6F). Placing channels further away from the soma progressively reduced spike height, similar to recorded responses. However, observed values were not matched in the simulated voltage offset. Even our most distal placement on the reconstructed morphology (Fig 6C) resulted in large spikes and small voltage offsets (Fig 6H).

The unreconstructed part of the aCC axon in the 3rd instar extends a variable distance (from 100 μm to 1 mm) based on body segment in which it is located (Fig 3A). To mimic this, we added to the morphology an axon compartment with proximal and distal sections with diameters of 0.8 and 0.7 μm, respectively. The lengths of these two sections were variable and only the distal section contained the NaT/Ks channels and a leak parameter of *g*_leak, ext_ = 120 μS/cm^2^, which allowed us to test placement of channels at different distances from the soma. A recapitulation of spike amplitude and voltage offset, to *in vivo* response, (Fig 6J) was generated by setting the proximal segment to 70 μm (Fig 6D). Although the length of the distal compartment was set at 500 μm in this instance, its exact value did not significantly affect the results (tested lengths 200 μm, 400 μm and 800 μm). Instead, the distance from the soma was critical for spike amplitude and offset: placing NaT/Ks channels further than 70 μm resulted in smaller spike amplitudes and larger voltage offsets. These results are summarized by comparing voltage offset (Fig 6K) and spike amplitude (Fig 6L) across the different placement scenarios of the NaT/Ks channels. The predicted SIZ location is no surprise considering the large voltage attenuation (10%) we observed in the passive morphology when a high frequency current (representative of spike currents) was injected at the SIZ location and measured at the somatic electrode (Table 3). However, physiologically this was unexpected because, based previous on experiments [15], the SIZ of insect neurons is proposed to be located on the main neurite just distal to where it exits the neuropil (filled arrow in Fig 3A). Our prediction places the SIZ much further distal, at about where the primary neurite exits the nerve cord and enters the periphery towards the muscle field (empty arrow in Fig 3A).

We did not attempt to find a precise match for the delay responses in this exercise: delays were not dependent on SIZ distance and could be obtained with an isopotential model by providing the right current injection value (see Fig 2C). To compare different channel distributions, channel density, kinetics, and voltage-dependence parameters were adjusted to bring the model neuron into the spiking regime in each scenario (Table 4). However, these adjustments alone were insufficient to match the voltage offset or spike amplitude features, which were primarily controlled by the anatomical parameters. A prediction of the location of the SIZ and therefore NaT channels is important because changes in different features of sodium currents are implicated in *Drosophila* seizure mutants [19, 20]. Having established the approximate location of the SIZ with reasonable confidence, we addressed the extent to which distally-generated sodium currents are distorted when they are recorded at the soma.

### Transient Na^+^ current filtering explains difference observed in heterologous expression

We have shown above that with a distal SIZ, the fast NaT current is filtered more than slower currents like Ks and the similarly non-inactivating NaP. A heavily-filtered (i.e., diminishing) NaT propagating to the soma would increase the observed NaP/NaT ratio, which may explain the unusually high NaP/NaT ratio recorded from larval motoneuron somata, with an average of 20% in wildtype flies [19]. In contrast, when DmNa_v_ is heterologously expressed in more compact cells, such as in *Xenopus laevis* oocytes, the largest NaP/NaT ratio observed was only 9.5% [22]. This two-fold difference between recordings of the same channel in different systems may be caused by the different morphologies of the two host cells. To test this hypothesis, we used the present model to simulate the effect of morphological location on Na^+^ current components.

**Fig 7:**
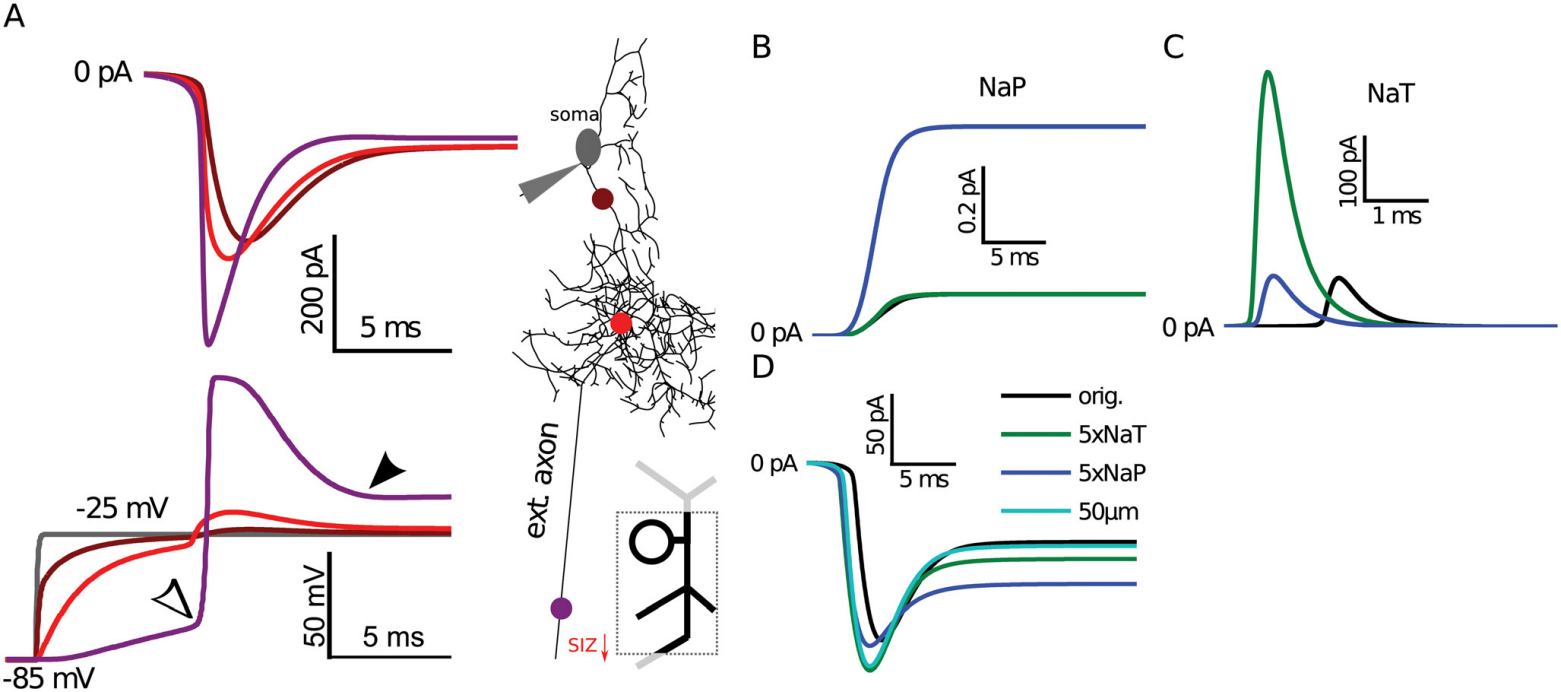
NaT sodium current component was filtered more than NaP in the simulated morphology. (**A**) Total axial sodium currents (top) recorded at several color-coded locations on the main neurite (axon) in response to voltage clamp. Voltage traces of the selected locations (bottom) showed that the quality of voltage clamp weakens distally. Model NaP (**B**) and NaT (**C**) current components recorded where they originated in the extended axon SIZ segment for in the original model (black), in models where the NaT (green) or NaP (blue) component was increased 5-fold. (**D**) Clamp currents recorded at the soma for the same three conditions and in a model where the SIZ was 20 μm closer to the soma (50 μm).

By simulating voltage clamp in our model, with the protocol used in previous sodium current recordings (Fig 1B), we confirmed that sodium currents generated at the SIZ are heavily filtered and therefore the apparent NaP/NaT ratio is increased as the current waveform propagates to the recording site at the soma (Fig 7A). In this simulation, there was a considerable decrease in the NaT peak amplitude, and therefore the NaP/NaT ratio at the SIZ was 55pA/230pA = 23%, while at the soma it increased to 65pA/145pA = 44%: a two-fold increase similar to that found between heterologous and endogenous recordings. However, this model was unable to replicate the experimentally observed endogenous NaP/NaT ratio of 20% with any combination of NaT and NaP conductance values. These simulations also revealed a result that is inconsistent with passive attenuation expected from filtering: at the soma, instead of being reduced, the apparent NaP was increased from 55 to 65 pA (Fig 7A).

To investigate the reason underlying the unexpected increase of NaP, we simulated the change in the total sodium currents under isolated conditions. First, we confirmed that when either the NaT or NaP conductance was increased 5-fold independently at the SIZ, a corresponding increase was observed at the soma in respective components (local measurements in Fig 7B and 7C respectively labeled as “5x NaT” and “5x NaP” in Fig 7D). These independent NaP and NaT manipulations were strong enough to increase excitability in a silent model, by causing it to reach firing rates of 136 and 181 Hz, respectively. Although small, an increase in somatic NaP current was observed with independent manipulation of NaT. However, somatic NaP remained unaffected when the model SIZ was moved 20 μm closer to the soma (marked with “50 μm” distance in Fig 7D), which resulted in as much increase of NaT at the soma as the five-fold increase of NaT at the SIZ. Another possibility of how NaT contributes to NaP is through its window current that results from incomplete inactivation in a small range of membrane potentials. Although the applied holding potential at the soma was −25 mV, the SIZ escaped from voltage clamp and reached −10 mV (at filled arrow in Fig 7A). At this membrane potential, the NaT conductance is almost completely inactivated and unlikely to result in any significant current (see Fig 1F). Thus, we conclude that the increase in NaP must be caused by filtering of the larger current in the NaT manipulation. In summary, we showed that the increased NaP/NaT observation *in vivo* could be attributed to membrane filtering attenuation of the NaT peak current. This is the result of the distal morphological location of the SIZ, which would be lacking when DmNa_v_ is heterologous expressed in oocytes.

## Discussion

The fruitfly, *Drosophila melanogaster*, provides a powerful model system for understanding the role of ion channels in both the development and function of the CNS. More recently, it has been successfully exploited to understand the connection of ion channels with many human neurological diseases. However, interrogation of ion channel function using traditional electrophysiological techniques, whilst possible, remains challenging in this insect because of the distal localization of spiking currents. Computational modeling provides a viable complement for this and many other applications, but is presently limited by a lack of complete models of identified neurons [11, 25, 26]. Here, we construct a model of an identified larval motoneuron—aCC—to demonstrate an approach to study distally-located ion channels. This approach consists of several steps: (1) Initial estimation of ion channel biophysics in space clamped structures (e.g., heterologous expression, or earlier developmental stages), (2) Constructing a detailed neuron model with these ion channels by using *in vivo* firing characteristics as a morphological signature to estimate location and distribution of channels (i.e., distal spike-generating currents), (3) Using the final model to estimate individual ion current contributions to firing activity and to quantify parameters of membrane artifacts on isolated channel recordings. We present several models of increasing complexity that are suitable to make these neurophysiological predictions. By modeling the likely distribution of active conductances, we surprisingly find that the SIZ is located very distally from the cell soma, at a point along the primary axon as it leaves the CNS to enter the muscle field. This extended physical distance, together with the dendritic branch points on the primary neurite, create the electrotonic distance and axial resistance that match aCC’s *in vivo* output. The membrane between the SIZ and soma strongly filters currents with fast activation kinetics and provides an explanation for the unusually high persistent to transient Na^+^ channel component ratio observed at the soma [19] compared to when the same Na^+^ channels are expressed in more compact cells; e.g., in *Xenopus* oocytes [22]. In addition, we predict that K^+^ channels are likely to be low density in most of the neuronal structure except where co-localized with sodium channels at the SIZ. These model predictions aid correcting for recording errors of both Na^+^ and K^+^ channel currents from *in vivo* recordings and guide future experiments to confirm their true locations. Furthermore, the method proposed in this paper to estimate location and accuracy of ionic current recordings is general and can be applied to any neuron as long as reasonable estimates of channel biophysics, *in vivo* spiking, and morphological details are known. In particular, our prediction that spiking currents are strongly low-pass filtered applies to other invertebrate neurons with distal SIZ like those found in Aplysia [43], crab [44] and leech [34].

### Semi-automated tuning of aCC channel biophysics and model parameters

In this study, we focused on aCC motoneurons from abdominal segments 2–4, although slight segmental differences exist [45]. When the channels we characterized were simulated together in the isopotential model, the model matched aCC’s observed firing rate and delay properties, which validated the accuracy of the estimated channel biophysics. We only included essential spiking currents in this model for simplicity. These were transient (NaT) and persistent (NaP) Na^+^, and transient (fast, Kf) and non-inactivating (slow, Ks) K^+^ currents. The Ks current (likely Shab) that we characterized activated at higher potentials (see Methods; effective *V*_1/2_ = ∼ 20 mV) than non-inactivating K^+^ channels found in the adult *Drosophila* MN5 identified motoneuron [27], which suggested that larval and adult motoneurons have different channel properties. We did not characterize Ca^2+^ currents in aCC—although some biophysical data existed [15, 18], it was insufficient to characterize Ca^2+^-dependent K^+^ (CaK) currents. CaK channels are important because they generate large outward currents dependent on calcium influx caused by Ca^2+^ channels. When Ca^2+^ currents were blocked using Cd^2+^, aCC motoneurons still fire action potentials, but the lack of Ca^2+^ currents may have other impacts on the results presented here. In particular, our prediction that the somatodendritic regions have small outward currents (i.e., Ks), also likely includes CaK currents. As expected from control experiments with Ca^2+^ currents, the firing rate properties of the model aCC would likely change if large CaK currents were co-localized with spiking currents. Adding these currents into the model will be important before simulating synaptic inputs onto the dendrites and may affect our estimate of SIZ location.

Consistent with the current magnitudes observed in aCC, the spiking mechanism in the model required larger Ks currents than NaT currents. This is unusual compared to several other invertebrate neuron models that have smaller K^+^ currents than Na^+^ currents [46, 47]. Since the ratio of these currents in the present models affects our results about channel distributions over the morphology, a more thorough exploration of the parameter space must be undertaken in the future. In the current state of the art, model parameter tuning is automated to produce a set of models, often called “ensemble of models”, which can then be taken to further analysis [48–50]. In contrast, the parameters in the three models presented here were obtained by a semi-automated hand-tuning procedure. One reason for this is the difference in the organism modeled: as opposed to specimens collected from the wild that may have large variations in neurophysiological properties [51, 52], *Drosophila* larvae used in this study are isogenetic, being bred from the same Canton-S wildtype genetic background, and therefore we might expect the motoneurons of these flies to have less variable channel conductances. A technical reason for not employing an ensemble model parameter search was the lack of an existing canonical model of aCC. A canonical model is often established through hand-tuning and reduces the parameter space to be explored for generating ensembles (e.g., by only varying maximal conductance density parameters around it). For building a canonical aCC model, biophysical properties of ionic currents had to be characterized, which resulted in our model having many more parameters than can be efficiently searched using automated methods. Without proper analysis and tuning of biophysical parameters, a conductance-space ensemble modeling effort would be premature. Therefore, semi-automated methods were used to determine some of the biophysical and morphological parameters (see Methods). Hand-tuning was used to establish the maximal conductance parameters to introduce the baseline canonical models of aCC. Having reduced the size of the parameter search space, these models open the way for applying ensemble neuron modeling methods to study robustness of the models’ behaviors to changes in parameters for neuron morphology, channel conductances, and biophysics. Furthermore, this canonical model’s parameters can be refined to match recordings from individual aCC motoneurons.

### Predicted spike initiation zone with high-density Na^+^ and K^+^ channels

Mammalian neurons are known, in some instances, to utilize active currents throughout their structure to aid signal transmission from distal dendritic tips [53]. However, our prediction of a compact morphology may explain why active amplification is only needed at significant distances from the soma in some invertebrate neurons. In some of these neurons, spikes are generated as far as several centimeters away from the soma [44]. Somatic and proximal regions of these neurons are often represented as either passive structures, or at least, without spiking currents [32]. Electrotonic compactness of our aCC model also results from its passive morphological parameters together with a low density of non-inactivating K^+^ currents in its proximal dendritic fields, allowing even the most distal dendritic inputs to reach the soma and reach part of the primary neurite, where we predict that the SIZ starts. However, the electrotonic distance was much larger for high frequency (100Hz, representative of spiking activity) stimuli, and it was the primary reason for the amplitude attenuation that occurred when spikes generated at the predicted SIZ location were observed at the soma. Our model SIZ region extends to the distal tip of the axon and channels are distributed homogeneously. The axon initial segment (AIS) characterized in mammals is shorter and contains a high density of Na^+^ channels, located more proximal than myelinated sections [54] where lower channel densities are sufficient for spike propagation. Consistent with the possibility of a shorter SIZ, simulations varying our model aCC’s SIZ length did not affect its predicted starting point. We predict it starts close to the root of the intersegmental nerve, where glia-sheathed nerve bundles (fascicles) start [55] that aid spike propagation, similar to myelin in mammals.

We predict that the SIZ starts 70 μm distal to the last branch point in our model aCC neuron. Elsewhere, in *Drosophila* mushroom body neurons, anatomical markers identified an AIS-like section on the primary neurite [56]. This region started about 25 μm away from the last dendritic branch point on the axon and was about 15 μm long. In terms of channel expression, a SIZ was also characterized in the adult *Drosophila* MN5 motoneuron [57]. Using an antibody, Na^+^ channel clustering was observed at about 100 μm distance from the soma along the primary neurite, consistent with our own prediction. The location of the SIZ on adult MN5 was about 10 μm distal to the last dendritic branch point, shorter than the prediction in our larval aCC model. However, the distance of the SIZ from the soma cannot be directly compared between MN5 and aCC because of differences in developmental stage and motoneuron type. In aCC, our modeling technique made a very precise prediction of SIZ localization. This prediction can be tested by future experiments using antibody labeling of DmNa_v_ channels (as in [57]), or with genetic modifications that affect anchoring of those channels at the SIZ. Any experiment modifying to the shape of DmNa_v_ currents and measuring them at the soma would also be a test of the modeling predictions. In fairness, several factors may challenge our prediction and influence the true location of the SIZ. Estimating spatial properties and channel biophysics are co-dependent, which makes their estimation a chicken-egg problem. Furthermore, channels, activity, and morphology were collected from different preparations with slightly varying conditions. These conditions can be studied further using automated parameter search methods and constructing ensembles of models as suggested above.

Finally, localizing the aCC SIZ allowed us to more realistically estimate the true appearance of ionic currents at their source. Even though space clamp errors and membrane filtering properties are known [38] and established in neuronal recordings, their effects are often not quantified [1, 2]. The method we propose here is general and can be applied to any neuron, with the provision that good estimates of channel biophysics are first measured. If there is no good initial estimate of the biophysics, one can potentially iterate through estimates of the biophysics and morphological tuning to reach a steady state. Having established the SIZ for aCC, it was especially important to understand whether the differences in recordings from highly varying spatial configurations of endogenously versus heterologously expressed DmNa_v_ channels were caused by the same underlying sodium current. It is often possible that additional proteins or subunits must be expressed to ensure faithfulness of heterologous expression [58]. Our results affirm that the difference is likely caused by filtering in the intact neuron as opposed to compact oocytes and that it is highly likely that both organisms are expressing the identical current. This is important in supporting *Xenopus* oocytes as an expression system for studying *Drosophila* DmNa_v_ channels. Our model was unable to replicate the exact endogenous NaP/NaT ratio observed in aCC, which points to a limitation of the current model construction. We suspect that this may be caused by space clamp artifacts and related to leak subtraction as these parameters affected the NaP/NaT ratio as well.

By constructing a morphological motoneuron model, with essential ion channel biophysics and distribution, we pave the way for predicting and testing genetic manipulations of ion channels with much better accuracy, which are an integral part of the study of *Drosophila* neuronal circuits. Recent advances at the macro level succeeded at automated reconstruction of these circuits [59, 60] and complete locomotion models [61]. Both necessitate our approach of specifying active electrical properties. Our prediction of the SIZ location in the presented model is a critical step in enabling us to address future questions about the effect of both distal and proximally generated currents. As our model is one of the few attempts for modeling morphological structure together with ion channel distribution of an invertebrate neuron (e.g., see [34, 62]), it can also be used as a good example of why these models can be useful. Our canonical aCC model can also be adapted to other similar *Drosophila* and other insect motoneurons [13], and even maybe to other invertebrate neurons as well based on the common features such as bipolar dendritic structure, soma as an offshoot of the main neurite, and distal SIZ. The model provides a new tool to address fundamental issues in neuronal function such as how excitability changes with channel regulation and modulation, and how distributions of dendritic channels affect synaptic integration. These issues can be addressed with the model by executing a vast number of simulations to exhaustively scan available channel and morphological parameter combinations that would complement and guide biological experiments, which are much more difficult and time consuming to execute.

## Methods

### Electrophysiology

Whole cell voltage- and current-clamp recordings were made from Canton-S wildtype *Drosophila* larvae as previously described [19, 63]. To measure biophysical channel properties, various ionic currents were isolated by chemical block using combinations of tetrodotoxin (TTX), tetraethylammonium chloride (TEA), and 4-aminopyridine (4-AP). Larvae were dissected differently based on developmental stage [15, 64, 65]. *Xenopus laevis* oocytes were used for heterologous expression of sodium channel constructs and recorded from with two-electrode voltage clamp method as described in [22].

### Model channel parameters

Channel currents were formalized using Hodgkin-Huxley (HH) type equations in the form of:

$$\begin{array}{c} I=\bar{g}m^{p}h\left(V_{m}-E\right)\;, \end{array}$$

where $\bar{g}$ is the maximal conductance, *m* and *h* are respectively activation and inactivation variables, *p* is the power of the activation gate, *V*_*m*_ is the membrane potential, and *E* is the reversal potential of the ion conducted through the channel. The *m* and *h* variables are each described with the differential equation for *x* below:

$$\begin{array}{c} \frac{dx}{dt}=\left(x_{\infty }\left(V_{m}\right)-x\right)/\tau_{x}\left(V_{m}\right)\;. \end{array}$$

The voltage-dependent steady-state, *x*_∞_(*V*_*m*_), is defined as a standard Boltzmann function:

$$\begin{array}{ccc} x_{\infty }\left(V_{m}\right) & = & \frac{1}{1+\exp((V_{m}-V_{1/2,x})/k_{x})}\;, \end{array}$$ (1)

where *V*_1/2, *x*_ and *k*_*x*_ are the activation half-voltage and slope factor in mV, respectively. For the voltage dependence parameters in *x*_∞_(*V*_*m*_) and the time constant, *τ*_*x*_(*V*_*m*_), functions for all channels, see Table 5.

**Table 5:** Channel biophysical parameters of Hodgkin-Huxley type models.

	Activation				Inactivation		
Current	*p*	$V_{\frac{1}{2},m}$(mV)	*k*_*m*_(mV)	*τ*_*m*_(ms)	$V_{\frac{1}{2},h}$(mV)	*k*_*h*_(mV)	*τ*_*h*_(ms)
NaT	3	−29.13	−8.92	$0.13+3.43/\left(1+\exp\left(\frac{V_{m}+45.35}{5.98}\right)\right)$	−47	5	$0.36+\exp\left(\frac{V_{m}+20.65}{-10.47}\right)$
NaP	1	−48.77	−3.68	1	–	–	–
Ks	4	−12.85	−19.91	$2.03+1.96/\left(1+\exp\left(\frac{V_{m}-29.83}{3.32}\right)\right)$	–	–	–
Kf	4	−17.55	−7.27	$1.94+2.66/\left(1+\exp\left(\frac{V_{m}-8.12}{7.96}\right)\right)$	−45	6	$1.79+515.8/\left(1+\exp\left(\frac{V_{m}+147.4}{28.66}\right)\right)$

The isopotential model’s channel parameters were previously published [20] and differ from there only slightly in NaT current time constant functions and voltage dependence of inactivation.

**Na^+^ channels.** We obtained parameters (see Table 5) for the above HH model for the transient component of the sodium channel (NaT) using recordings reported by [66]. These were whole-cell recordings from embryonic neurons maintained in culture. The reversal potential and maximal conductance were reported as *E*_Na_ = 45 mV and ${\bar{g}}_{\text{Na}}=500\;\text{pS/pF}$. For a whole-cell capacitance *C*_*m*_ = 20 pF of a 3rd instar motoneuron this becomes ${\bar{g}}_{\text{Na}}=10\;\text{nS}$. The inactivation *V*_1/2_ and the gating charge (*z*) were also reported. To find the *k*_*x*_ in Eq (1) from the gating charge, we used the equation *k*_*x*_ = *k*_*B*_ *T*/*ze*, where *k*_*B*_ *T*/*e* = 25.4 J/C is a physical constant that contains the Boltzmann constant (*k*_*B*_), absolute temperature in K (*T*), and electronic charge (*e*) [67]. The activation *V*_1/2_ and slope factor were measured using the I-V plot in Fig 2B of [66].

We estimated the inactivation properties by fitting a sigmoidal function to the measured inactivation time constant. The activation time constant was obtained from the measured time-to-peak (*t*_*p*_), by using the equation *τ*_*m*_ = ((*τ*_*m*_+*τ*_*h*_)/*τ*_*m*_−*exp*(*t*_*p*_/*τ*_*m*_))^2^. The fits were obtained in MATLAB (Mathworks). The data of O'Dowd and Aldrich [66] was matched with the resulting channel current simulation (Fig 1A). Isolated sodium currents recorded in 3rd instar aCC motoneurons (Fig 1B) were not used for fitting this model because of large onset delays that are likely caused by difficulty of space clamping distal currents.

The HH parameters of the persistent component of the sodium channel (NaP) were obtained from heterologous expression of the *Drosophila* *paralytic* (*DmNav*) gene transcripts in *Xenopus laevis* oocytes [22]. A slow voltage ramp protocol was used to isolate NaP and a model of the endogenous Ca^2+^-dependent Cl^-^current (*I*_ClCa_) in oocytes [68] was fitted to be subtracted from the current response (Fig 1C).

**Simultaneous fits for fast (Kf) and slow (Ks) currents.** We modeled both K^+^ current components using the same HH formalism; except that Ks had no inactivation gate. Two sets of voltage-clamp recordings were used that have differential prepulse (200 ms) holding levels: −90 mV and −10 mV, respectively. A holding level of −10 mV inactivates the fast (Kf) component [69]. We fitted the parameters of both currents simultaneously, including their activation and inactivation properties [70]. Instead of using the time solution of the HH equations for ideal voltage step inputs, we fully simulated channels to follow the imperfectly changing voltage levels of the voltage clamp protocols, such that experimental conditions (including passive electrode artifacts) are replicated in simulations. In these simulations, partially inactivated Kf still contributed to the output. After the holding step, the protocol steps to variable levels between −20 and +40 mV with 10 mV steps for 50 ms. The fits to both protocols are shown superimposed (Fig 1D). A semi-automated method was used to fit parameters. A non-linear least squares fitter (lsqcurvefit function) in MATLAB was used with the large-scale trust-region reflective Newton method. The 95% confidence intervals of individual fits were observed and bad fits were rejected. The parameter fitting algorithm obeyed physiologically realistic ranges for each parameter and simulated with a general ordinary differential equation solver using a custom MATLAB toolbox. We used a separate voltage clamp protocol where holding levels were varied to estimate the inactivation curve of the Kf channel (Fig 1E). The fitted parameters for all channels were used to plot activation and time constant voltage dependence graphs (Fig 1F).

### Isopotential and two-compartment models

The isopotential and two-compartment models were constructed and simulated using the XPPAut software [71] using the Euler numerical integration method with a step size of 0.001 ms. The passive membrane and active channel parameters of both models were manually tuned and their robustness was confirmed (Table 6). For the isopotential model, the membrane voltage *V*_*m*_ was calculated with the differential equation d*V*_*m*_/d*t* = −(*I*_Ks_ + *I*_Kf_ + *I*_NaT_ + *I*_NaP_ + *g*_leak_(*V*_*m*_ − *E*_leak_) − *I*_stim_)/*C*_*m*_, where the active channels are defined as above (Table 5) and *I*_stim_ is the external stimulus current. Voltages are in mV, currents in pA, and conductances in nS. For the two-compartment model, the somatic membrane voltage, *V*_*m*_, was calculated with d*V*_*m*_/d*t* = −(*I*_Ks_ + *I*_Kf_ + *g*_leak_(*V*_*m*_ − *E*_leak_) − *I*_stim_ + *g*_axon_(*V*_*m*_ − *V*_*a*_))/*C*_*m*_, where *g*_axon_ is the coupling conductance in nS. The axon compartment voltage, *V*_*a*_, was calculated with d*V*_*a*_/d*t* = − (*I*_Ks_ + *I*_Kf_ + *I*_NaT_ + *I*_NaP_ + *g*_aleak_(*V*_*a*_ − *E*_leak_) + *g*_axon_(*V*_*a*_ − *V*_*m*_))/*C*_*a*_. To simplify tuning of the passive model parameters to match recorded responses of firing rate, voltage offset, and spike amplitude features in the two-compartment model, a constant input conductance parameter, *g*_*i*_, was introduced. We defined it as *g*_*i*_ = 0.47 nS to match the observed voltage offsets in response to current stimulation, which left only the *g*_leak_ and *g*_axon_ parameters to be tuned. The axonal leak parameter was then calculated from these variables as $g_{\text{aleak}}={\left({\left(g_{i}-g_{\text{leak}}\right)}^{-1}-g_{\text{axon}}^{-1}\right)}^{-1}$. The holding current for stimulus protocols started at −6.5 pA.

**Table 6:** Maximal conductance (*g*), reversal potential (*E*), and capacitance (*C*) parameters of the isopotential and two-compartment models.

Isopotential model			Two-compartment model					
			(soma)			(axon)		
*g*_Ks_ =	50	nS	*g*_Ks_ =	1	nS	*g*_Ks_ =	700	nS
*g*_Kf_ =	24.1	nS	*g*_Kf_ =	1	nS	*g*_Kf_ =	200	nS
*E*_K_ =	-80	mV	*E*_K_ =	-80	mV	*E*_K_ =	-80	mV
*g*_NaT_ =	100	nS	*g*_leak_ =	0.05	nS	*g*_NaT_ =	180	nS
*g*_NaP_ =	0.8	nS	*E*_leak_ =	-55	mV	*g*_NaP_ =	0.01	nS
*g*_leak_ =	6.8	nS	*C* =	10	pF	*g*_leak_ =	0.63	nS
*E*_leak_ =	-55	mV	*g*_axon_ =	1.3	nS	*E*_leak_ =	-55	mV
*C* =	4	pF				*C* =	1.8	pF

### Morphologically reconstructed model

A stack of confocal images was obtained from a 3rd instar aCC motoneuron 48hr after hatching (Fig 3B) using methods described previously [31]. Morphological reconstruction from the image stack was obtained using Amira (Visage Imaging GmbH, Berlin, Germany, http://vsg3d.com). The reconstructed morphology is publicly available at the NeuroMorpho.org online repository for neuronal morphologies (http://neuroMorpho.org/neuroMorpho/neuron_info.jsp?neuron_name=aCC-L3-motoneuron). Thin dendrites may be missing or their diameters may be slightly overestimated because of the limits of light microscopy.

The electrical structure of the morphological model neuron was constructed and simulated with the Neuron simulator, Release 7.1 ([72]; http://www.neuron.yale.edu). To import the morphological reconstruction into the Neuron simulator, the morphometric information was converted using the software tools neuroConstruct ([73]; http://www.neuroconstruct.org/) and NeuronLand (http://www.neuronland.net/NL.html) (James Ogden).

To estimate electrophysiological measurement artifacts, we modeled electrode and circuit parameters that are not compensated by the recording amplifier (Fig 3C). We fitted the passive parameters of this circuit to five passive voltage-clamp protocol recordings from a late 3rd instar aCC motoneuron (Table 2). A representative fit is shown (Fig 3D). Passive properties of the model were fit following a procedure similar to the method defined in [74] using Neuron’s “multiple-run fitter” algorithm.

Using the resulting passive parameters, gross statistics of different sections of the morphology were calculated as single compartments with equivalent surface area (Table 1). This method does not maintain the length constant, which requires the morphology to obey strict rules [75]. To maintain surface area, we scaled the diameters of compartments by their length. This made the values for *R*_*i*_ slightly inaccurate, but values of *C*_*m*_ more accurate. The input impedance of the model was calculated with a simulation method using the Impedance Frequency Tool in Neuron (see Fig 4C). Capacitance of a similar 3rd instar neuron was experimentally determined to be 20.0 pF, which is slightly larger than the total capacitance (16.91 pF) that can be achieved from this morphological reconstruction. We attribute this difference to slight differences in morphology of the cells and limited fidelity of the reconstruction process.

Once the passive parameters were determined, Neuron’s CellBuilder component was used to insert active conductances into the morphology. For simulations, default numerical integration parameters of Neuron were used, including ionic reversals (*E*_Na_ = 65 mV and *E*_K_ = −74 mV). The leak reversal was selected as −85 mV. For estimating K^+^ channel distributions, potential averages from spiking traces were calculated without removing individual spikes. The model neuron is available both in the ModelDB repository (accession number 152028) and in the Open Source Brain (OSB) initiative (identified as drosophila-acc-l3-motoneuron-gunay-et-al-2014).

To measure distal currents in the morphology, voltage differences between nearby compartments were recorded and divided by internal resistivity parameters. For measuring active currents with this method using voltage clamp, subtraction of capacitance and leak artifacts was needed like in neuronal recordings. This was achieved by running two simulations: one with and one without active channels and subtracting the measured currents between the two. This method, like in biological experiments, assumed that voltage clamp conditions are identical between the two simulations. Therefore, Na^+^ currents were not recorded exactly at the SIZ because of large space clamp artifacts, but instead in the proximal compartment of the extended axon.

## References

[1] Williams SR, Mitchell SJ (2008) Direct measurement of somatic voltage clamp errors in central neurons. Nat Neurosci 11: 790–798. 10.1038/nn.2137 18552844

[2] Hartline DK, Castelfranco AM (2003) Simulations of voltage clamping poorly space-clamped voltage-dependent conductances in a uniform cylindrical neurite. J Comput Neurosci 14: 253–269. 10.1023/A:1023208926805 12766427

[3] Golowasch J, Marder E (1992) Ionic currents of the lateral pyloric neuron of the stomatogastric ganglion of the crab. J Neurophysiol 67: 318–331. 1373762 10.1152/jn.1992.67.2.318

[4] Olsen SR, Wilson RI (2008) Cracking neural circuits in a tiny brain: New approaches for understanding the neural circuitry of *Drosophila* . Trends Neurosci 31: 512–520. 10.1016/j.tins.2008.07.006 18775572 PMC2845908

[5] Salkoff L, Baker K, Butler A, Covarrubias M, Pak M, et al (1992) An essential set of K(+) channels conserved in flies, mice and humans. Trends Neurosci 15: 161–166. 10.1016/0166-2236(92)90165-5 1377421

[6] Solc C, Aldrich R (1988) Voltage-gated potassium channels in larval CNS neurons of *Drosophila* . J Neurosci 8: 2556–2570. 3249242 10.1523/JNEUROSCI.08-07-02556.1988 PMC6569521

[7] Tsunoda S, Salkoff L (1995) Genetic-analysis of *Drosophila* neurons—shal, shaw, and shab encode most embryonic potassium currents. J Neurosci 15: 1741–1754. 7891132 10.1523/JNEUROSCI.15-03-01741.1995 PMC6578176

[8] Islas L, Sigworth F (1999) Voltage sensitivity and gating charge in Shaker and Shab family potassium channels. J Gen Physiol 114: 723–741. 10.1085/jgp.114.5.723 10539976 PMC2230542

[9] Covarrubias M, Wei A, Salkoff L (1991) Shaker, Shal, Shab, and Shaw express independent K+ current systems. Neuron 7: 763–773. 10.1016/0896-6273(91)90279-9 1742024

[10] Peng IF, Wu CF (2007) Differential contributions of Shaker and Shab K+ currents to neuronal firing patterns in *Drosophila* . J Neurophysiol 97: 780–794. 10.1152/jn.01012.2006 17079336

[11] Herrera-Valdez MA, McKiernan EC, Berger SD, Ryglewski S, Duch C, Crook S (2013) Relating ion channel expression, bifurcation structure, and diverse firing patterns in a model of an identified motor neuron. J Comput Neurosci 34: 211–229. 10.1007/s10827-012-0416-6 22878689 PMC6595220

[12] Marder E (2011) Variability, compensation, and modulation in neurons and circuits. Proc Natl Acad Sci U S A 108 Suppl 3: 15542–15548. 10.1073/pnas.1010674108 21383190 PMC3176600

[13] Choi J, Park D, Griffith L (2004) Electrophysiological and morphological characterization of identified motor neurons in the *Drosophila* third instar larva central nervous system. J Neurophysiol 91: 2353–2365. 10.1152/jn.01115.2003 14695352

[14] Wolfram V, Baines RA (2013) Blurring the boundaries: developmental and activity-dependent determinants of neural circuits. Trends Neurosci 36(10): 610–619. 10.1016/j.tins.2013.06.006 23876426 PMC3794160

[15] Baines R, Bate M (1998) Electrophysiological development of central neurons in the *Drosophila* embryo. J Neurosci 18: 4673–4683. 9614242 10.1523/JNEUROSCI.18-12-04673.1998 PMC6792699

[16] Rohrbough J, Broadie K (2002) Electrophysiological analysis of synaptic transmission in central neurons of *Drosophila* larvae. J Neurophysiol 88: 847–860. 12163536 10.1152/jn.2002.88.2.847

[17] Baines R, Uhler J, Thompson A, Sweeney S, Bate M (2001) Altered electrical properties in *Drosophila* neurons developing without synaptic transmission. J Neurosci 21: 1523–1531. 11222642 10.1523/JNEUROSCI.21-05-01523.2001 PMC6762927

[18] Worrell JW, Levine RB (2008) Characterization of voltage-dependent Ca2+ currents in identified *Drosophila* motoneurons in situ. J Neurophysiol 100: 868–878. 10.1152/jn.90464.2008 18550721 PMC2525733

[19] Marley R, Baines RA (2011) Increased persistent Na+ current contributes to seizure in the slamdance bang-sensitive *Drosophila* mutant. J Neurophysiol 106: 18–29. 10.1152/jn.00808.2010 21451059 PMC3129721

[20] Lin WH, Günay C, Marley R, Prinz AA, Baines RA (2012) Activity-dependent alternative splicing increases persistent sodium current and promotes seizure. J Neurosci 32: 7267–77. 10.1523/JNEUROSCI.6042-11.2012 22623672 PMC3400946

[21] George AL (2005) Inherited disorders of voltage-gated sodium channels. J Clin Invest 115(8): 1990–1999. 10.1172/JCI25505 16075039 PMC1180550

[22] Lin WH, Wright DE, Muraro NI, Baines RA (2009) Alternative splicing in the voltage-gated sodium channel DmNa(v) regulates activation, inactivation, and persistent current. J Neurophysiol 102: 1994–2006. 10.1152/jn.00613.2009 19625535 PMC2746785

[23] Song J, Tanouye MA (2008) From bench to drug: Human seizure modeling using *Drosophila* . Prog Neurobiol 84: 182–191. 10.1016/j.pneurobio.2007.10.006 18063465 PMC2267866

[24] Turrigiano G, Nelson S (2004) Homeostatic plasticity in the developing nervous system. Nat Rev Neurosci 5: 97–107. 10.1038/nrn1327 14735113

[25] Kidd JF, Sattelle DB (2006) The effects of amyloid peptides on A-type K+ currents of *Drosophila* larval cholinergic neurons: Modeled actions on firing properties. Invert Neurosci 6: 207–213. 10.1007/s10158-006-0034-y 17106756

[26] Gouwens NW, Wilson RI (2009) Signal propagation in *Drosophila* central neurons. J Neurosci 29: 6239–6249. 10.1523/JNEUROSCI.0764-09.2009 19439602 PMC2709801

[27] Ryglewski S, Duch C (2009) Shaker and Shal mediate transient calcium-independent Potassium current in a *Drosophila* flight motoneuron. J Neurophysiol 102: 3673–3688. 10.1152/jn.00693.2009 19828724 PMC2804405

[28] Ryglewski S, Kilo L, Duch C (2014) Sequential acquisition of cacophony calcium currents, sodium channels and voltage-dependent potassium currents affects spike shape and dendrite growth during postembryonic maturation of an identified *Drosophila* motoneuron. Eur J Neurosci 39(10): 1572–1585. 10.1111/ejn.12517 24620836 PMC4433752

[29] Landgraf M, Bossing T, Technau G, Bate M (1997) The origin, location, and projections of the embryonic abdominal motorneurons of *Drosophila* . J Neurosci 17: 9642–9655. 9391019 10.1523/JNEUROSCI.17-24-09642.1997 PMC6573408

[30] Nicolaï LJJ, Ramaekers A, Raemaekers T, Drozdzecki A, Mauss AS, et al (2010) Genetically encoded dendritic marker sheds light on neuronal connectivity in *Drosophila* . Proc Natl Acad Sci U S A 107: 20553–20558. 10.1073/pnas.1010198107 21059961 PMC2996714

[31] Zwart MF, Randlett O, Evers JF, Landgraf M (2013) Dendritic growth gated by a steroid hormone receptor underlies increases in activity in the developing *Drosophila* locomotor system. Proc Natl Acad Sci U S A 110: E3878–E3887. 10.1073/pnas.1311711110 24043825 PMC3791713

[32] Soto-Treviño C, Rabbah P, Marder E, Nadim F (2005) Computational model of electrically coupled, intrinsically distinct pacemaker neurons. J Neurophysiol 94: 590–604. 10.1152/jn.00013.2005 15728775 PMC1941697

[33] Ball JM, Franklin CC, Tobin AE, Schulz DJ, Nair SS (2010) Coregulation of ion channel conductances preserves output in a computational model of a crustacean cardiac motor neuron. J Neurosci 30: 8637–8649. 10.1523/JNEUROSCI.6435-09.2010 20573909 PMC4473856

[34] Tobin AE, Van Hooser SD, Calabrese RL (2006) Creation and reduction of a morphologically detailed model of a leech heart interneuron. J Neurophysiol 96: 2107–2120. 10.1152/jn.00026.2006 16760352 PMC2897741

[35] Hendrickson EB, Edgerton JR, Jaeger D (2011) The capabilities and limitations of conductance-based compartmental neuron models with reduced branched or unbranched morphologies and active dendrites. J Comput Neurosci 30: 301–321. 10.1007/s10827-010-0258-z 20623167 PMC3058356

[36] Landgraf M, Thor S (2006) Development and structure of motoneurons. Int Rev Neurobiol 75: 33–53. 10.1016/S0074-7742(06)75002-4 17137922

[37] Schaefer J, Worrell J, Levine R (2010) Role of intrinsic properties in *Drosophila* motoneuron recruitment during fictive crawling. J Neurophysiol 104: 1257–66. 10.1152/jn.00298.2010 20573969 PMC2944697

[38] Spruston N, Jaffe DB, Johnston D (1994) Dendritic attenuation of synaptic potentials and currents: The role of passive membrane properties. Trends Neurosci 17: 161–166. 10.1016/0166-2236(94)90094-9 7517596

[39] Kim H, Jones KE, Heckman CJ (2014) Asymmetry in signal propagation between the soma and dendrites plays a key role in determining dendritic excitability in motoneurons. PLoS One 9: e95454 10.1371/journal.pone.0095454 25083794 PMC4118843

[40] Rall W (1977) Core conductor theory and cable properties of neurons. In: Handbook of Physiology, American Physiological Society, volume 1. pp. 39–97.

[41] Hartline DK, Gassie DV, Jones BR (1993) Effects of soma isolation on outward currents measured under voltage clamp in spiny lobster stomatogastric motor neurons. J Neurophysiol 69: 2056–2071. 7688800 10.1152/jn.1993.69.6.2056

[42] Schaefer AT, Helmstaedter M, Schmitt AC, Bar-Yehuda D, Almog M, et al (2007) Dendritic voltage-gated K+ conductance gradient in pyramidal neurones of neocortical layer 5B from rats. J Physiol 579: 737–752. 10.1113/jphysiol.2006.122564 17158172 PMC2151356

[43] Tauc L (1962) Site of origin and propagation in spike in the giant neuron of Aplysia. J Gen Physiol 45: 1077–1097. 10.1085/jgp.45.6.1077 13919850 PMC2195234

[44] Meyrand P, Weimann JM, Marder E (1992) Multiple axonal spike initiation zones in a motor neuron: Serotonin activation. J Neurosci 12: 2803–2812. 1613558 10.1523/JNEUROSCI.12-07-02803.1992 PMC6575837

[45] Srinivasan S, Lance K, Levine RB (2012) Segmental differences in firing properties and potassium currents in *Drosophila* larval motoneurons. J Neurophysiol 107: 1356–1365. 10.1152/jn.00200.2011 22157123 PMC3311690

[46] Prinz AA, Billimoria CP, Marder E (2003) Alternative to hand-tuning conductance-based models: Construction and analysis of databases of model neurons. J Neurophysiol 90: 3998–4015. 10.1152/jn.00641.2003 12944532

[47] Lamb DG, Calabrese RL (2013) Correlated conductance parameters in leech heart motor neurons contribute to motor pattern formation. PLoS One 8: e79267 10.1371/journal.pone.0079267 24260181 PMC3832487

[48] Prinz AA (2010) Computational approaches to neuronal network analysis. Philos Trans R Soc Lond B Biol Sci 365: 2397–2405. 10.1098/rstb.2010.0029 20603360 PMC2894951

[49] Marder E, Taylor AL (2011) Multiple models to capture the variability in biological neurons and networks. Nat Neurosci 14: 133–138. 10.1038/nn.2735 21270780 PMC3686573

[50] Günay C (2014) Neuronal model databases In: Jaeger D, Jung R, editors, Encyclopedia of Computational Neuroscience, Springer URL http://www.springerreference.com/docs/html/chapterdbid/348230.html.

[51] Bucher D, Prinz AA, Marder E (2005) Animal-to-animal variability in motor pattern production in adults and during growth. J Neurosci 25: 1611–1619. 10.1523/JNEUROSCI.3679-04.2005 15716396 PMC6725924

[52] Schulz D, Goaillard J, Marder E (2006) Variable channel expression in identified single and electrically coupled neurons in different animals. Nat Neurosci 9: 356–362. 10.1038/nn1639 16444270

[53] Edgerton JR, Hanson JE, Günay C, Jaeger D (2010) Dendritic sodium channels regulate network integration in Globus Pallidus neurons: A modeling study. J Neurosci 30: 15146–15159. 10.1523/JNEUROSCI.2662-10.2010 21068320 PMC3022226

[54] Kole MHP, Ilschner SU, Kampa BM, Williams SR, Ruben PC, et al (2008) Action potential generation requires a high sodium channel density in the axon initial segment. Nat Neurosci 11: 178–186. 10.1038/nn2040 18204443

[55] Rodrigues F, Schmidt I, Klämbt C (2011) Comparing peripheral glial cell differentiation in *Drosophila* and vertebrates. Cell Mol Life Sci 68: 55–69. 10.1007/s00018-010-0512-6 20820850 PMC11114915

[56] Trunova S, Baek B, Giniger E (2011) Cdk5 regulates the size of an axon initial segment-like compartment in mushroom body neurons of the *Drosophila* central brain. J Neurosci 31: 10451–10462. 10.1523/JNEUROSCI.0117-11.2011 21775591 PMC3150738

[57] Kuehn C, Duch C (2012) Putative excitatory and putative inhibitory inputs are localised in different dendritic domains in a *Drosophila* flight motoneuron. Eur J Neurosci. 10.1111/ejn.12104 23279094 PMC3604049

[58] Derst C, Walther C, Veh RW, Wicher D, Heinemann SH (2006) Four novel sequences in *Drosophila melanogaster* homologous to the auxiliary *para* sodium channel subunit TipE. Biochem Biophys Res Commun 339: 939–948. 10.1016/j.bbrc.2005.11.096 16325765

[59] Cardona A, Saalfeld S, Preibisch S, Schmid B, Cheng A, et al (2010) An integrated micro- and macroarchitectural analysis of the *Drosophila* brain by computer-assisted serial section electron microscopy. PLoS Biol 8 10.1371/journal.pbio.1000502 20957184 PMC2950124

[60] Tsechpenakis G, Mukherjee P, Kim MD, Chiba A (2012) Three-dimensional motor neuron morphology estimation in the *Drosophila* ventral nerve cord. IEEE Trans Biomed Eng 59: 1253–1263. 10.1109/TBME.2011.2181166 22203698

[61] Gjorgjieva J, Berni J, Evers JF, Eglen SJ (2013) Neural circuits for peristaltic wave propagation in crawling *Drosophila* larvae: Analysis and modeling. Front Comput Neurosci 7: 24 10.3389/fncom.2013.00024 23576980 PMC3616270

[62] Takashima A, Hikosaka R, Takahata M (2006) Functional significance of passive and active dendritic properties in the synaptic integration by an identified nonspiking interneuron of crayfish. J Neurophysiol 96: 3157–3169. 10.1152/jn.00680.2006 16914611

[63] Wolfram V, Southall TD, Brand AH, Baines RA (2012) The Lim-homeodomain protein Islet dictates motor neuron electrical properties by regulating K(+) channel expression. Neuron 75: 663–674. 10.1016/j.neuron.2012.06.015 22920257 PMC3427859

[64] Marley R, Baines RA (2011) Dissection of first- and second-instar *Drosophila* larvae for electrophysiological recording from neurons: the flat (or fillet) preparation. Cold Spring Harb Protoc 2011. 10.1101/pdb.prot065649 21880809

[65] Marley R, Baines RA (2011) Dissection of third-instar *Drosophila* larvae for electrophysiological recording from neurons. Cold Spring Harb Protoc 2011. 10.1101/pdb.prot065656 21880808

[66] O'Dowd D, Aldrich R (1988) Voltage-clamp analysis of sodium-channels in wild-type and mutant *Drosophila* neurons. J Neurosci 8: 3633–3643. 2848103 10.1523/JNEUROSCI.08-10-03633.1988 PMC6569590

[67] Hodgkin A, Huxley A (1952) A quantitative description of membrane current and its application to conduction and excitation in nerve. J Physiol 117: 500–544. 10.1113/jphysiol.1952.sp004764 12991237 PMC1392413

[68] Barish M (1983) A transient calcium-dependent chloride current in the immature *Xenopus* oocyte. J Physiol-London 342: 309–325. 10.1113/jphysiol.1983.sp014852 6313909 PMC1193960

[69] Wu CF, Haugland FN (1985) Voltage clamp analysis of membrane currents in larval muscle fibers of *Drosophila*: Alteration of potassium currents in Shaker mutants. J Neurosci 5: 2626–2640. 2413182 10.1523/JNEUROSCI.05-10-02626.1985 PMC6565151

[70] Willms A, Baro D, Harris-Warrick R, Guckenheimer J (1999) An improved parameter estimation method for Hodgkin-Huxley models. J Comput Neurosci 6: 145–168. 10.1023/A:1008880518515 10333160

[71] Ermentrout B (2002) Simulating, analyzing, and animating dynamical systems: A guide to XPPAUT for researchers and students. Philadelphia, PA: Society for industrial and Applied Mathematics (SIAM).

[72] Carnevale N, Hines M (2006) The NEURON Book. Cambridge, UK: Cambridge University Press.

[73] Gleeson P, Steuber V, Silver RA (2007) NeuroConstruct: A tool for modeling networks of neurons in 3D space. Neuron 54: 219–235. 10.1016/j.neuron.2007.03.025 17442244 PMC1885959

[74] Segev I, Burke RE (1998) Compartmental models of complex neurons. In: Koch and Segev [76], chapter 3, pp. 93–136.

[75] Rall W, Agmon-Snir H (1998) Cable theory for dendritic neurons. In: Koch and Segev [76], chapter 2, pp. 27–92.

[76] Koch C, Segev I, editors (1998) Methods in neuronal modeling: From synapses to networks. Cambridge, MA: MIT, 2nd edition.

